# Reproductive functions and fertility preservation in men with sickle cell disease

**DOI:** 10.1111/andr.70021

**Published:** 2025-03-28

**Authors:** Clarisse Leblanc, Nathalie Sermondade, Diane Rivet‐Danon, Ludmilla Aworet‐Ogouma, Anna Ly, Guillaume Bachelot, Françoise Lionnet, Aline Santin, Anne‐Gael Cordier, Kamila Kolanska, Rachel Lévy, Isabelle Berthaut, Charlotte Dupont

**Affiliations:** ^1^ Service de Biologie de La Reproduction‐CECOS Hôpital Tenon, APHP, Sorbonne Université Paris France; ^2^ INSERM, UMR, Centre de Recherche St‐Antoine, CRSA Sorbonne Université Paris France; ^3^ Service de Médecine Interne Centre de Référence Syndromes Drépanocytaires Majeurs Hôpital Tenon, APHP. Sorbonne Université Paris France; ^4^ Service de Gynécologie Obstétrique et Médecine de la Reproduction Hôpital Tenon, AP‐HP, Sorbonne Université Paris France

**Keywords:** fertility preservation, hydroxyurea, male fertility, semen parameters, sickle cell disease

## Abstract

**Background:**

Sickle cell disease (SCD) is a prevalent hereditary disorder with significant morbidity, including potential impacts on male fertility. This study aims to evaluate the semen parameters in men with SCD and assess the outcomes of fertility preservation strategies.

**Methods:**

This retrospective study included 121 men with SCD referred to the fertility Centre at Tenon University Hospital, Paris, between 2012 and 2023. Patients were categorized into three groups based on hydroxyurea (HU) exposure: without HU (WHU), ongoing HU(OHU), and previous HU (PHU). Clinical and semen parameters data were collected and compared with those of 107 healthy sperm donors. Semen parameters were analyzed according to World Health Organization guidelines, and sperm freezing protocols were standardized. Statistical analysis was performed to compare semen parameters between groups.

**Results:**

Of the 121 patients, 117 successfully collected semen. All semen parameters, including volume, concentration, total count, motility, vitality, and morphology, were significantly reduced in SCD patients without HU exposure compared to donors. Nine had azoospermia and 45 had oligozoospermia, compared to 11 sperm donors with oligozoospermia (*p* < 0.05). The impact of HU on semen parameters could not be demonstrated due to the small‐sample size. Fertility preservation outcomes showed a mean of 1.96 collections per patient, yielding a mean of 8.7 straws, with a majority requiring in vitro fertilization with intracytoplasmic sperm injection (ICSI) for future use. Seven patients used their cryopreserved sperm, resulting in two successful births.

**Conclusions:**

This study, the largest of its kind, confirms significant alterations in semen parameters in men with SCD. Due to deleterious effects of treatments on male reproductive functions, fertility preservation remains crucial for these patients. Further research is needed to refine fertility preservation strategies and address the long‐term reproductive health of men with SCD.

## 1 INTRODUCTION

Sickle cell disease (SCD) is one of the most prevalent hereditary disorders worldwide, predominantly affecting populations in sub‐Saharan Africa, India, the Mediterranean basin, and the Middle East.^1^ Each year, approximately 300,000 children are born with severe forms of SCD.^2^, ^3^

SCD is an autosomal recessive genetic disorder characterized by a mutation in the beta‐globin gene (HBB), where a single nucleotide substitution (adenine to thymine) in codon 6 results in the replacement of glutamic acid with valine. This mutation produces abnormal hemoglobin (Hb), known as sickle cell hemoglobin or hemoglobin S (HbS).^4^ HbS exhibits reduced solubility and increased polymerization under low oxygen conditions, leading to the deformation of red blood cells into a characteristic sickle shape. These deformed cells exhibit altered rheological properties and reduced lifespan, contributing to the pathophysiology of the disease.^5^

SCD manifests clinically with hemolytic anemia and painful vaso‐occlusive crises that can affect various organs, including the cardiovascular, cerebrovascular, and renal systems.^4^ Importantly, SCD can also impair reproductive functions and fertility. In men, recurrent hypoxic–ischemic events and chronic inflammation associated with SCD can detrimentally affect testicular function and sperm production.^6^ The limited studies available suggest alterations in semen parameters in men with SCD; however, these studies often have small‐sample sizes and methodological limitations.^7^, ^8^, ^9^, ^10^, ^11^, ^12^, ^13^ Notably, the most recent and largest study to date presented conflicting results regarding these alterations.^14^

Advancements in treatments have significantly improved the prognosis of SCD, leading to increased life expectancy and enabling more men with the condition to reach reproductive age.^15^, ^16^ Consequently, considerations of fertility and reproductive health have become increasingly important for these patients. However, the treatments such as hydroxyurea (HU), which are designed to alleviate SCD symptoms and enhance patient quality of life, may also impact male reproductive functions.^8^, ^9^, ^13^, ^17^

Given the potential effects of both SCD and its treatments on male fertility, it is crucial to incorporate fertility considerations into the comprehensive management of young men with SCD. A thorough understanding of how the disease and its treatments affect reproductive functions is essential to provide appropriate guidance and, if necessary, offer fertility preservation options.

The objective of this study was to compare semen parameters of men with SCD to those of healthy control men (sperm donors) and to evaluate the effectiveness of fertility preservation strategies offered to these patients.

## 2 MATERIALS AND METHODS

### 2.1 Patient selection

This retrospective study focused on men with SCD referred to the fertility Centre at Tenon University Hospital, Paris, between 2012 and 2023. Patients were referred by their physicians (internists, hematologists, endocrinologists, general practitioners) for information about fertility preservation and, in most cases, to benefit from sperm cryopreservation. In France, fertility preservation is guided by established medical protocols and legal frameworks. French public health insurance system covers medical fertility preservation procedures, ensuring accessibility for patients undergoing treatments that may affect fertility. Currently, there are no national recommendations for referring patients with SCD to fertility preservation consultations. Patients are generally referred before starting HU or undergoing HSCT. A medical prescription was necessary for sperm freezing. Patients were offered medical consultations followed by an appointment for sperm collection and freezing.

Exclusion criteria included a prior history of gonadotoxic treatment unrelated to SCD or any condition that could have adversely affect spermatogenesis.

### 2.2 Data collection

The healthcare pathway of men with SCD after their first consultation at the study center was fully documented. Data regarding age, previous and current treatments, biomarkers of chronic hemolysis (Hb and lactate dehydrogenase (LDH)), ferritin, medical history in the 5 years preceding fertility preservation, reproductive history, semen parameters, and sperm cryopreservation were collected. Patients were classified into three groups based on HU intake: without hydroxyurea (WHU) patients had no history of HU intake, ongoing hydroxyurea (OHU) patients were treated with HU at the time of fertility preservation, and previous hydroxyurea (PHU) patients were on a washout period at the time of fertility preservation.

Clinical and biological data from sperm donors recruited to the public study center during the same period were also collected to compare semen parameters between the groups. The sperm donors were healthy men aged 18–44 years old, with or without children.

The study protocol was approved by a local ethics committee (IRB SALF—number 20234) on 11 December 2023.

### 2.3 Semen parameters analysis

Semen samples were collected by masturbation into a sterile plastic cup in the laboratory. Samples were allowed to liquefy at room temperature for 30 min and conventional semen quality was evaluated according to the World Health Organization's (WHO) 2010 and 2021 guidelines.^18^ Semen volume was measured by weighing the collection container before and after ejaculation, with the difference in weight corresponding to the volume, assuming a density of 1 g/mL. Sperm concentration was determined using a Malassez chamber, where a diluted semen sample was placed. At least 200 sperm cells were counted under a microscope to calculate concentration. Sperm motility was assessed twice by placing 10  µl of semen between two microscope slides and coverslips; the samples were then examined under a microscope to evaluate the percentage of motile spermatozoa. The eosin‐nigrosin staining technique was employed to assess sperm vitality. Two hundred spermatozoa were assessed. Sperm morphology was evaluated using the modified David classification following Harris–Schorr staining.^19^ The analyses were carried out by trained technicians and the laboratory is accredited under the iso15189 standard.

Oligozoospermia, or a decrease in total sperm count, was defined as fewer than 39 million spermatozoa in the ejaculate according to WHO 2021 guidelines.

Only semen parameters from the first semen collection were considered for statistical analysis. All samples from patients and sperm donors were collected at the same center and analyzed under the same conditions.

### 2.4 Sperm freezing

Semen samples from men with SCD and sperm donors were frozen according to the same standardized protocol. The samples were diluted with cryoprotectant medium (SpermFreezTM, FertiPro NV, Belgium) and distributed into sperm straws (SpermFreezeTM, FertiPro, Beernem, Belgium). They were frozen in liquid nitrogen vapor using an automatic freezer (Nano‐Digitcool, Cryo Bio System). The straws were then plunged into liquid nitrogen and stored in nitrogen tanks. Freezing tolerance was evaluated after one straw had been thawed. Motility and sperm concentration were analyzed, and the total number of progressive motile spermatozoa per straw (NMSPS) was calculated. A possible assisted reproductive technology (ART) strategy was defined according to NMSPS as follows: straws containing fewer than 1 million progressive motile spermatozoa were considered usable for In vitro fertilisation (IVF) with intracytoplasmic sperm injection (ICSI), and straws containing more than 1 million progressive motile spermatozoa were considered usable for intrauterine insemination (IUI).

### 2.5 Statistical analysis

Semen parameters were compared between men with SCD WHU and sperm donors, and between men with SCD WHU and those previously treated with PHU or with OHU. Variables are presented as mean ± standard error of measurement (SEM) for quantitative variables and as a percentage for qualitative variables. Quantitative variables were analyzed using an independent *t*‐test or Wilcoxon–Mann–Whitney test when appropriate, and Fisher's exact test for qualitative variables. All statistical analyses were performed using Prism 9 software (GraphPad Software Inc., La Jolla, CA, USA), and *p* < 0.05 was considered significant.

## 3 RESULTS

### 3.1 Description of the population

**FIGURE 1:**
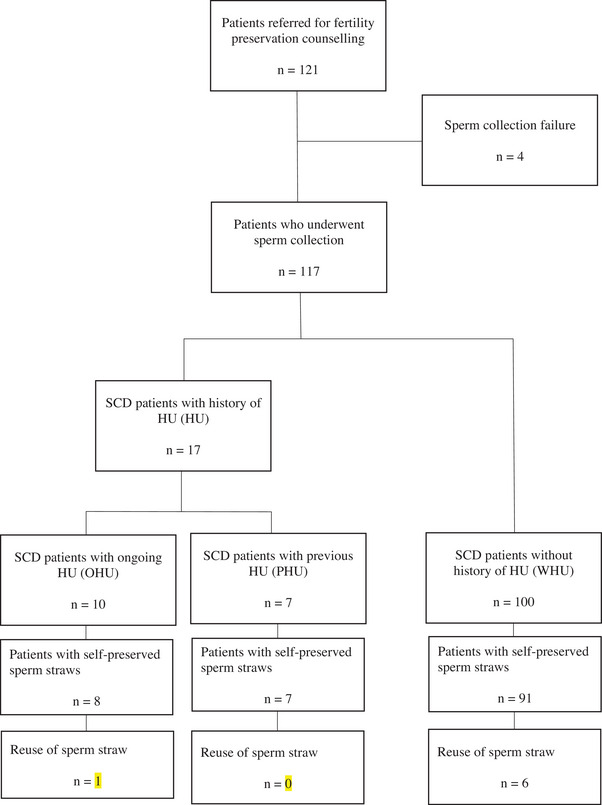
Flowchart of the study population.

Between January 2012 and November 2023, 121 SCD patients were referred for fertility preservation counseling appointments. One hundred and seventeen patients attempted at least one semen collection. Four patients experienced semen collection failure. Of the 117 patients who successfully collected sperm, 100 had never been exposed to HU, 10 were being treated with HU at the time of fertility preservation, and 7 were in a washout period. Among them, sperm cryopreservation was successfully achieved in 91, 8, and 7 patients, respectively (Figure 1).

Markers of chronic hemolysis (Hb and LDH), ferritin, current treatments, duration and doses, SCD complications, reproductive history and planned treatments are presented in Table 1. All men who had children at the time of fertility preservation had conceived spontaneously with their partners.

**TABLE 1:** Clinical characteristics of SCD patients.

	Without HU		Ongoing HU		Previous HU	
	*n*	Mean ± SEM or *n* (%)	*n*	Mean ± SEM or *n* (%)	*n*	Mean ± SEM or *n* (%)
Age (years)	100	28.0 ± 0.8	10	29.8 ± 2,7	7	26.9 ± 3.2
HU						
‐ Age at HU start (years) ‐ Dose (mg/kg/day) ‐ Dose (mg/day) ‐ Exposure time (days) ‐ Duration of washout period (months)	/ / / /	/ / / /	6 7 7 6	16.5 ± 3.7 18.1 ± 1.3 1321 ± 130.4 3787.5 ± 828.6 /	4 3 3 4 5	17.3 ± 5.3 12.2 ± 0.7 866.7 ± 133.3 2998.8 ± 1452.1 9 ± 3.8
Biomarkers						
‐ Hemoglobin (g/dL) ‐ LDH (U/L) ‐ Ferritin (µg/L)	69 61 39	9.3 ± 0.1 510.1 ± 29.4 258.6 ± 51.9	9 8 5	9.0 ± 0.4 516 ± 104.3 207.8 ± 95.2	6 5 3	9.3 ± 0.8 521.2 ± 119.1 149.0 ± 84.9
Regular blood transfusion (%) ‐ frequency	70 5	5 (7.1%) Monthly	8 3	3 (37.5%) Monthly	6 1	1 (16.7%) Every two months
Complications	98		10		7	
‐ V‐OC ‐ ATS ‐ Cholecystectomy		100 (100%) 65 (65%) 60 (60%)		9 (90%) 7 (70%) 4 (40%)		7 (100%) 3 (42.9%) 3 (42.9%)
Children Men with children before freezing		22 (26.2%)		3 (30%)		3 (50%)
‐ Number of children per man (median (min–max))	84	2 (1–5)	10	1 (1–2)	6	1 (1–3)
Planned treatment	100		10		10	
‐ none ‐ HU ‐ HSCT		1 (1.0%) 93 (94.9%) 4 (4.1%)		0 9 (90%) 1 (10%)		0 6 1

*Note*: Data are presented as mean ± SEM or *n* (%).

Abbreviations: SCD, sickle cell disease patients; HU, hydroxyurea; *n*, number of data available; LDH, lactate dehydrogenase; V‐OC, Vaso‐occlusive crisis; ATS, acute thorax syndrome.

### 3.2 Comparison of semen parameters between sickle cell disease men and sperm donors

#### 3.2.1 hydroxyurea patients versus sperm donors

SCD men without HU exposure were significantly younger than the sperm donors (28.0 ± 0.8 vs. 34.7 ± 0.6; *p* < 0.0001). All semen parameters, including volume, sperm concentration, total sperm count, progressive motility, vitality, and sperm morphology, were significantly reduced in SCD patients compared to donors (Table 2). Moreover, 9% SCD patients WHU presented azoospermia and 45% presented oligozoospermia, whereas only 10.1% sperm donors presented oligozoospermia. This difference was statistically significant (Table 2).

**TABLE 2:** Conventional semen parameters values.

Parameters	Sperm donors	SCD without HU	p WHU vs. SD	Ongoing HU	p OHU vs. WHU	Previous HU	p PHU vs. WHU
*N*	107	100		10		7	
Age (years)	34.7 ± 0.6	28.04 ± 0.8	<0.0001	29.8 ± 2.7	NS	26.9 ± 3.7	NS
Abstinence (days)	4.2 ± 0.9	8.6 ± 0.99	0.0013	3.7 ± 0.7	NS	5.7 ± 4.7	NS
Volume (mL)	3.4 ± 0.15	2.9 ± 0.2	0.026	2.3 ± 0.6	NS	1.8 ± 0.7	NS
Sperm concentration (106/mL)	66.2 ± 6.1	26.1 ± 3.5	<0.0001	22.0 ± 10.0	NS	13.1 ± 4.4	NS
Sperm numeration (106)	223.1 ± 24.1	85.1 ± 13.9	<0.0001	65.5 ± 32.3	NS	36.3 ± 23.2	NS
Progressive motility (%)	48.9 ± 1.2	33.1 ± 1.8	<0.0001	35.3 ± 7.0	NS	29.5 ± 8.9	NS
Vitality (%)	62.9 ± 1.3	49.9 ± 2.1	<0.0001	43.9 ± 11,4	NS	43.6 ± 9.4	NS
Morphology (%)	10.5 ± 0.7	6.9 ± 0.8	0.0012	3.0 ± 1.3	NS	8.2 ± 3.8	NS
Azoospermia	0	9 (9%)	NA	2 (20%)	NS	0	NA
Oligozoospermia	11 (10.1%)	45 (45%)	<0.0001	4 (40%)	NS	5 (71.4%)	NS

*Note*: Data are presented as mean ± SEM or *n* (%). *p *< 0.05 was considered significant.

Abbreviations: SD, sperm donors; SCD, sickle cell disease patients; HU, hydroxyurea; WHU, without hydroxyurea; OHU, ongoing hydroxyurea; PHU, previous hydroxyurea; NS, non‐significant; NA, non‐applicable.

#### 3.2.2 hydroxyurea and previous hydroxyurea patients versus without hydroxyurea patients

Patients with a history of HU exposure were less numerous than those who had never been treated. Alteration in semen parameters was not statistically significant in SCD patients with ongoing or previous HU compared to SCD men without HU (Table 2).

### 3.3 Results of sickle cell disease's sperm preservation and straw use

In the context of fertility preservation, 91 patients, 8 OHU and 7 PHU patients had sperm freezing. They underwent a mean of 1.43–1.96 sperm collections, enabling the freezing of a mean of 7.3–8.7 sperm straws. The NMSPS ranged from 0.7 to 1.5 million, and the progressive motility after thawing was between 19.9% and 21.7%. The difference between groups was not significant. Possible ART strategies for cryopreserved sperm (IUI + ICSI vs. ICSI only) did not differ between groups, and in the majority of cases, only ICSI procedures would be possible (> 63.7%) (Table 3).

**TABLE 3:** SCD patients sperm cryoconservation characteristics.

	SCD without HU	Ongoing HU	*p* OHU vs. WHU	Previous HU	*p* PHU vs. WHU
Number of patients	91	8	NS	7	NS
Number of sperm collections	1.96 ± 0.1	1.63 ± 0.2	NS	1.43 ± 0.2	NS
Number of straws at first sperm collection	8.7 ± 0.7	7,3 ± 2.3	NS	8,1 ± 2.2	NS
NMSPS (106) at first sperm collection	1.5 ± 0.3	1.1 ± 0.5	NS	0.7 ± 0.2	NS
Progressive motility after thawing (%) at first sperm collection	19.9 ± 1,7	21.4 ± 6.2	NS	21.7 ± 7.3	NS
Possible ART strategy IIU + ICSI	33 (36,3%)	1 (12.5%)	NS	0	NA
ICSI	58 (63,7%)	7 (87.5%)		7 (100%)	

*Note*: Data are presented as mean ± SEM or n (%). *p *< 0.05 was considered significant.

Abbreviations: SCD, sickle cell disease patients; HU, hydroxyurea; WHU, without hydroxyurea; OHU, ongoing hydroxyurea; PHU, previous hydroxyurea; IUI, intrauterine insemination; ICSI, intracytoplasmic sperm injection; NMSPS, number of progressive motile spermatozoa per straw; NS, non‐significant; NA, non‐applicable.

Of the patients who had cryopreserved sperm, seven returned for parental project with using their frozen samples (Figure 1). Four patients benefited from ICSI cycles in our center, while three patients went to another fertility center. Among the patients treated in our center, two have had a healthy child and ART is ongoing for two couples. One child was conceived using sperm freezed during the washout period (PHU).

## 4 DISCUSSION

This study, which includes the largest cohort of patients to date, confirms the significant alterations of semen parameters in men presenting with SCD independently of the treatment by HU. These men are at a higher risk of oligozoospermia and azoospermia when compared to a control population. The mechanisms behind this phenomenon are likely numerous and complex, possibly beginning as early as childhood. A link has been established between SCD and a decreased spermatogonial reserve in prepubertal children with SCD.^20^, ^21^ This decrease could be attributed to subclinical testicular vaso‐occlusive episodes, asymptomatic infarcts, and testicular perfusion deficits resulting from chronic anemia. In adults, local ischemia secondary to vaso‐occlusive crises, coupled with a higher risk of chronic fatigue, inflammation, and infection, may also contribute to impaired semen parameters.^6^

Improved care for SCD patients in resource‐rich countries has increased their life expectancy, allowing more patients to reach adulthood.^15^, ^16^ Consequently, reproductive issues in these men must be addressed early, providing them with information and, if necessary, tailored fertility preservation options. While there is no longer any debate about the alteration of semen parameters in men with SCD, the evolution of these parameters over time remains unknown. A progressive deterioration in semen parameters may occur in conjunction with the patient's health status. Furthermore, treatments for vaso‐occlusive crises and hematopoietic stem cell transplantation (the only curative treatment) impact male reproductive functions.^8^, ^9^, ^13^, ^17^, ^22^ Currently, there are no standardized guideline to assist professionals in this area. A multidisciplinary team of professionals has been assembled in France to address these issues and develop standardized practices at the national level. Nevertheless, in prepubertal boys, fertility preservation options are limited, with only immature testicular tissue freezing available.^23^ However, this procedure remains invasive and experimental, and there is currently no option for utilization.^24^ Therefore, only young boys undergoing hematopoietic stem cell transplantation should be proposed with immature testicular tissue freezing, because of highly gonadotoxic conditioning treatments. In postpubertal young men, sperm freezing following ejaculation is the standard procedure and should be prioritized. It must be offered before initiating conditioning treatments for hematopoietic stem cell transplantation. Regarding HU, due to the lack of proof of its safety on sperm DNA integrity and the uncertain reversibility after treatment discontinuation, it is recommended to offer sperm freezing before starting treatment or to initiate a washout period for 3 months. However, the washout period is not always feasible, as observed in 10 patients in this study. The impact of HU on semen parameters could not be demonstrated in this study due to the small‐sample size, but it has been shown in other publications.^8^, ^9^, ^13^, ^17^ Considering only conventional semen parameters, fertility preservation seems to be possible in patients taking HU, since sperm freezing was achievable in 8 out of 10 patients. Regarding the teratogenic risk, published data on children conceived by men treated with hydroxycarbamide are scarce, but no specific malformative effects attributable to the paternal treatment have been reported to date. The authors report the births of healthy children.^9^, ^10^, ^12^, ^13^ Moreover, the child, from our cohort study, conceived using cryopreserved sperm while his father was undergoing HU treatment is also in good health.

In our series, sperm freezing was possible for the majority of patients. Altered semen parameters impact the quality of the sperm straws, as most contain few progressive motile spermatozoa, making ICSI the only viable option in many cases. The requirement of using ICSI is more demanding for the partner and can represent a significant cost in countries where ART are not covered by health insurance. It is also worth noting that the utilization rate of cryopreserved sperm remains low, but this should not undermine the relevance of fertility preservation strategies, especially as many patients are still very young. Finally, fertility preservation was not possible for a few men, either due to failure in semen collection or because of azoospermia. The relevance of testicular biopsy in these cases is therefore a consideration. Further studies will be necessary to integrate this strategy into the management of these patients.

This paper presents some limitations. Only the results of the first semen collection were considered for analysis, however, this approach ensured that all samples were analyzed under the same conditions, maintaining consistency across the study. Moreover, patients and controls were not matched for age or ethnicity and the retrospective nature of the study may limit the ability to control for all potential confounding factors. Besides, until now, most patients were referred prior to initiating treatment, at a stage when their disease was causing crises that could impact various systems, including reproductive functions. As a result, these findings may not be fully generalizable to all men with SCD, particularly those with milder symptoms. Also, further prospective studies will be needed to confirm our results, we here present the largest study to date evaluating the impact of SCD and its treatments on semen parameters, providing comprehensive data and robust statistical analysis. The inclusion of a control group of healthy sperm donors allows for a direct comparison of semen parameters, enhancing the validity of our findings.

Very little data are available on the fertility of men with SCD. However, the findings regarding semen parameters should encourage healthcare professionals to address the topic of sperm cryopreservation.

## 5 CONCLUSION

This study highlights the significant impact of SCD on male reproductive functions, confirming alterations in semen parameters and increased risks of oligozoospermia and azoospermia. Improved patient care has extended life expectancy, making fertility preservation an important consideration for men with SCD. While sperm freezing is feasible for most patients, challenges remain, particularly for those with azoospermia or collection failures. Further research is needed to refine fertility preservation strategies, including the potential use of testicular biopsies, to ensure comprehensive care and reproductive options for men with SCD.

## AUTHOR CONTRIBUTIONS

Clarisse Leblanc participated in the study conception and design, interpreted the data and drafted the manuscript. Nathalie Sermondade and Diane Rivet‐Danon participated in study conception and design, interpretation of the data, and critically revised the manuscript for intellectual content. Ludmilla Aworet‐Ogouma, Anna Ly, Guillaume Bachelot, Françoise Lionnet, Aline Santin, Anne‐Gael Cordier, Kamila Kolanska, and Rachel Lévy participated in acquisition of data, patient care, and critically revised of the manuscript for intellectual content. Isabelle Berthaut participated in study conception and design, interpretation of the data, and critically revised the manuscript for intellectual content. Charlotte Dupont supervised the study, participated in study conception and design, interpretation of the data, performed the statistical analysis, and drafted the manuscript.

## CONFLICT OF INTEREST STATEMENT

The authors declare no conflicts of interest.

## Data Availability

The data that support the findings of this study are available on request from the corresponding author. The data are not publicly available due to privacy or ethical restrictions.
